# Integrative analysis and machine learning on cancer genomics data using the Cancer Systems Biology Database (CancerSysDB)

**DOI:** 10.1186/s12859-018-2157-7

**Published:** 2018-04-24

**Authors:** Rasmus Krempel, Pranav Kulkarni, Annie Yim, Ulrich Lang, Bianca Habermann, Peter Frommolt

**Affiliations:** 1 Regional Computing Center of the University of Cologne (RRZK), Cologne, Germany; 2 Bioinformatics Facility, CECAD Research Center, University of Cologne, Cologne, Germany; 3 Institut de Biologie du Développement, Aix-Marseille University, Marseille, France; 4 Max Planck Institute for Biochemistry, Martinsried, Germany

## Abstract

**Background:**

Recent cancer genome studies on many human cancer types have relied on multiple molecular high-throughput technologies. Given the vast amount of data that has been generated, there are surprisingly few databases which facilitate access to these data and make them available for flexible analysis queries in the broad research community. If used in their entirety and provided at a high structural level, these data can be directed into constantly increasing databases which bear an enormous potential to serve as a basis for machine learning technologies with the goal to support research and healthcare with predictions of clinically relevant traits.

**Results:**

We have developed the Cancer Systems Biology Database (CancerSysDB), a resource for highly flexible queries and analysis of cancer-related data across multiple data types and multiple studies. The CancerSysDB can be adopted by any center for the organization of their locally acquired data and its integration with publicly available data from multiple studies. A publicly available main instance of the CancerSysDB can be used to obtain highly flexible queries across multiple data types as shown by highly relevant use cases. In addition, we demonstrate how the CancerSysDB can be used for predictive cancer classification based on whole-exome data from 9091 patients in The Cancer Genome Atlas (TCGA) research network.

**Conclusions:**

Our database bears the potential to be used for large-scale integrative queries and predictive analytics of clinically relevant traits.

**Electronic supplementary material:**

The online version of this article (10.1186/s12859-018-2157-7) contains supplementary material, which is available to authorized users.

## Background

Large-scale cancer genome studies based on Next-Generation Sequencing (NGS) technology have enabled extensive research on tumorigenesis and treatment rationales [14]. The amount of data that has been generated and made available contrasts its limited accessibility to the research community. There is an increasing demand for customized queries to the data in a way that is accessible to scientists and physicians without any knowledge in bioinformatics. Genomic data from studies in The Cancer Genome Atlas (TCGA) research network obtained through the Genomic Data Commons (GDC) Data Portal (https://portal.gdc.cancer.gov) are available for multiple molecular layers and are provided in formats processed through appropriate software packages for the analysis of the raw data for every data type. The size of these processed data is orders of magnitude smaller than the raw data, in particular for whole-genome sequencing experiments, but provided in a diverse range of file formats in which the data are variably well structured. Thus, it is particularly challenging to transform these file-based data into a structure which allows a technically reasonable way to integrate data obtained by multiple technologies with manually curated data recorded in a clinical context. This underlines the need for highly flexible database structures which are suitable to model data from TCGA studies, but are generic enough to also combine TCGA data with locally acquired data obtained in a clinical context.

We present here the newly developed Cancer Systems Biology Database (CancerSysDB) portal which allows integrated analyses across multiple data types and across multiple cancer cohorts from The Cancer Genome Atlas (TCGA) research network, but also from locally acquired data in a clinical context. With its current workflows, our system allows fast integrative analysis of whole-exome (WXS) and transcriptome (RNA-Seq) sequencing data. By making use of standardized JSON-based meta data formats, the CancerSysDB can be integrated into existing analysis workflows. The CancerSysDB enables highly structured organization of data from multi-OMICS technologies and makes them accessible for big data analytics on the entirety of all data ever processed on a particular site. Conceptually, this includes the prediction of clinically relevant parameters such as therapeutic response from existing pharmacogenomic data in the CancerSysDB.

## Methods

### Implementation

The CancerSysDB was written in Groovy on the Grails framework based on the JVM stack which bundles state-of-the-art web frameworks behind a simple interface. The CancerSysDB is a web application which needs a database instance and an application server and can run Linux shell scripts and other executables from a command line. The data source is behind a hibernate facade keeping the system independent from the database implementation used and the optimization in the background. The delivered versions are based on a docker file to automatically build an environment and run the database application for personal use. A demo instance can be used to make personalized queries to the database using publicly available TCGA data. The source code of the CancerSysDB is available on GitHub (https://github.com/RRZK/CancerSysDB).

The system can be configured to run in two different modes. The *public mode* can be used to query publicly available data without any login. The publicly available main instance of the CancerSysDB available on http://cancersys.uni-koeln.de is running in public mode and provides access to data on 11,410 patients from the Cancer Genome Atlas (TCGA) research network. This instance includes data on somatic mutations (based on WXS data), differential gene expression (based on comparative RNA-Seq analysis between tumors and tissue-derived normals), somatic copy number alterations (based on Affymetrix SNP 6.0 microarrays) as well as all clinically derived annotations of the TCGA patient data. These data types provide a powerful basis for arbitrary queries defined by the user. All TCGA data types provided through the CancerSysDB are open access data and can be obtained from the TCGA data portal without exclusive access. Users have to adhere to the TCGA data access policies that apply to these open access data (https://gdc.cancer.gov/access-data/data-access-policies). On the other hand, the *private mode* requires a login for any interaction. This mode is strongly recommended if you are working with restricted data. The University of Cologne is operating a *private mode* instance of the CancerSysDB for the organization of genomic data from in-house studies. It is used in combination with the recently published cancer genomics data processing workflow system *QuickNGS Cancer* [1] which extends our NGS bioinformatics suite *QuickNGS* [15] and allows highly scalable and standardized analysis of cancer NGS data with minimum hands-on analysis time. Various features of the CancerSysDB are compared to those of other cancer genome data integration tools in Table 1.

**Table 1:** Comparison of various features of the CancerSysDB with those of other cancer genomics data integration tools

	CancerSysDB	TCGAbiolinks	RTCGA	cBio portal
GUI	Web framework based on Groovy/Grails	Based on Shiny	None	Web framework based on Spring Java
Query schema	Hibernate	R scripting	R scripting	SQL
Data upload	Parametrized CSV file upload	Direct access to GDC through API	Data packages available on Bioconductor	CSV files plus meta file
Query definition	JSON-based	Combination of R commands	Combination of R commands	REST-based API
Portability	Native Docker implementation	Hosted on Bioconductor	Hosted on Bioconductor	Hosted on GitHub
Programming skills required	No	Yes	Yes	No

### Data model and queries

The maintainer of a CancerSysDB instance can describe the connection between data and the main structure of the application in JSON files to bring the context structure of data into the database. The database consists of four main data types:

- *Structural data* manages the patients and samples,
- *Molecular data* is derived from cancer genome analysis,
- *Clinical data* is associated to the clinical course of a patient’s disease,
- *Genomic annotation* provides information on genes and meta data about these genes.

The data model and principles how to develop database queries is further described on GitHub at https://github.com/RRZK/CancerSysDB/tree/master/web-app/data/Workflows . Data can be uploaded through the API or manually with the web front end. The API enables automated uploads from processing infrastructures like high performance computing (HPC) environments. A collection of Python scripts for upload automation is delivered with the database. We are using these scripts to link the analysis workflows on the *QuickNGS Cancer* pipeline to the CancerSysDB. The internal design of the web application empowers the maintainer to easily extend the data model, extend the import behavior and integrate custom data structures.

The maintainer of an instance of the CancerSysDB is provided with a fully controllable environment for the development of custom workflows. A custom workflow can be described in a JSON file and extended with analysis scripts and static data in a zip file which can be dynamically uploaded into the database (documentation available on the GitHub). The actual data is retrieved using queries written in the Hibernate Query Language (HQL) and the results of the queries are saved as CSV files in order to increase reproducibility on a dynamically updated database. Subsequent computations can rely on arbitrary executables in a Linux environment. The container architecture provides the encapsulation for the workflows. To control the command line based execution, packages and libraries can be installed on creation of the docker container or wrapped directly into the files to be executed by the workflow.

### Data preparation

All TCGA data were obtained as level 3 data from the Legacy Archive of The Cancer Genome Atlas (TCGA) data portal. Data on somatic mutations were based on whole-exome sequencing with MAF files obtained from the Firehose pipeline of the Genome Data Analysis Center (GDAC) at the Broad Institute. Data on somatic copy number alterations were based on the SNP 6.0 microarray platform (Affymetrix Inc., CA, USA) given as genomic segments of equal copy number derived from the Circular Binary Segmentation (CBS) algorithm [8]. For gene expression analysis, raw RNA-Seq read counts were re-processed and compared between tumor tissues and tissue-derived normal samples using version 1.21.1 of the DESeq2 algorithm and its implementation as an R package [6]. These tissue-derived normal controls are available from only a minority of the patients in TCGA, but we consider them more suitable for a comparative tumor/normal analysis than the blood-derived normals existing for most patients. The currently existing workflows were implemented using version 3.3.3 of the functional statistics language R (http://www.r-project.org). The random forest workflow was implemented with the R package ‘randomForest’, version 4.6–12.

## Results and discussion

In order to demonstrate how the CancerSysDB can help to obtain analysis results of immediate relevance for research projects or clinical prognosis, we showcase the analytical power by three example queries, by one machine learning workflow on the CancerSysDB and by an interactive workflow of visualizing mitochondrial pathways. The results of these showcases can be reproduced using the query and analysis source code provided in Additional file 1.

### TP53-dependent analysis of overall survival, genome stability, and mutation types

The tumor suppressor gene TP53 is the most frequently deleted and mutated gene across all tumor types [3]. In the TCGA cancer cohorts, its mutation rate is highly variable and ranges up to > 75% in some cancer types [16]. The CancerSysDB enables comparative genomic analyses of patients with and without mutations in TP53 by employing three different query workflows which we operate across > 11,000 patients from 33 TCGA studies.

**Fig. 1:**
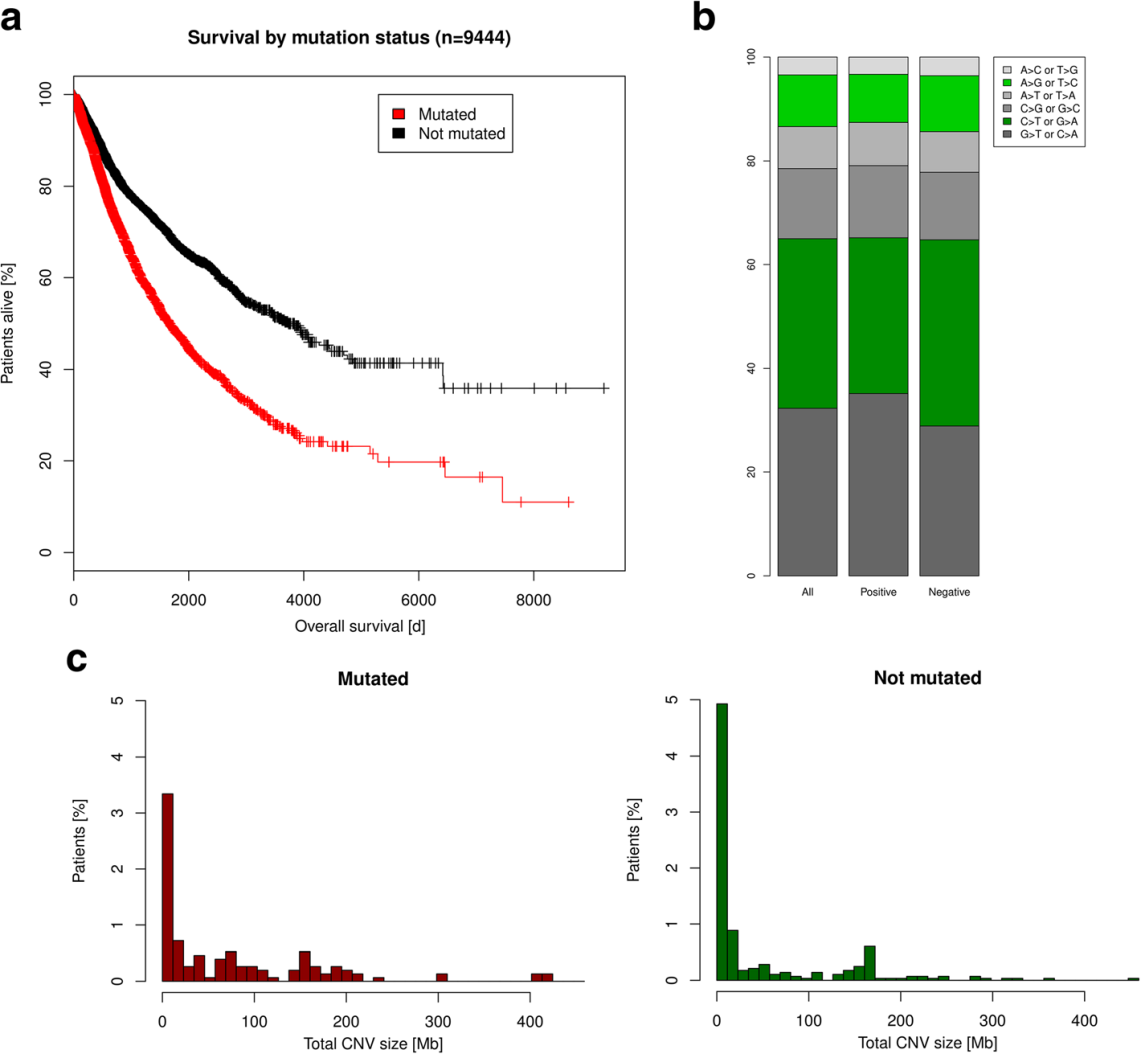
Analysis results for workflows splitting multiple TCGA cohorts into TP53-mutant and non-mutant patients: **a** Overall survival is significantly different between TP53-mutant (red curve) and non-mutant patients (black curve) with a more favorable for non-mutant patients (gain in median survival: 2066 days, *p* <  0.0001, *n* = 9444). **b** The distribution of the mutations types in lung adenocarcinoma is strongly shifted towards an increase of G > T transversions in TP53 mutant compared to non-mutant patients (*p* = 0.0006, *n* = 584). **c** Genomic stability is quantified in terms of the overall size of somatic copy number alterations (sCNA) compared between tumor and normal. sCNA are considered as genomic amplifications above a level of 3 and as genomic deletions below a level of 1 for the signal ratio between tumor and paired normal sample. The difference between TP53 mutant and non-mutant patients is highly significant in glioblastoma multiforme (*p* = 0.0132, *n* = 379)

- *Overall survival depending on mutation status:* Across all TCGA cohorts, patients with a mutation in TP53 show an unfavorable prognosis regarding overall survival compared to TP53 wild type patients (*p* <  0.0001, *n* = 9444; Fig. 1a; Table 2a).
- *Transversions and transitions depending on mutation status:* The somatic mutational landscape of patients with lung adenocarcinoma exhibits a significant shift towards G > T transversions when compared between patients with and without mutations in TP53 (*p* = 0.0006, *n* = 584; Fig. 1b; Table 2b). G > T transversions have been shown to be induced by oxidative stress in lung cancers of tobacco smokers [12]. Their enrichment in patients with mutated TP53 is likely caused by the impaired induction of apoptosis upon these exogenic damages.
- *Genomic complexity depending on mutation status:* Among the patients with glioblastoma multiforme, those with TP53 mutations are characterized by, on average, stronger genomic instability than the TP53 wild type patients (*p* = 0.0132, *n* = 379; Fig. 1c; Table 2c). This general loss of genomic stability in TP53-mutated patients can be attributed to the role of TP53 as a mediator of apoptosis in response to somatically acquired DNA damage of cancer cells and has been described in previous studies [7].

**Table 2:** Results of TP53-dependent analysis of genomic and clinical characteristics

(a)							
	Patients	Events	5-year survival rate [%]		Median survival		95% CI
TP53 mutant	3772	1237	47.4		1670		[1526; 1818]
TP53 non-mutant	5672	1128	66.9		3736		[3262; 4267]
(b)							
		Patients			CNAs [Mb]		
TP53 mutant		133			74.5		
TP53 non-mutant		246			50.5		
(c)							
		TP53			ATM		
VarType	All	Mutant [%] (*n* = 320)	Non-mutant [%] (*n* = 265)	*p*-value	Mutant [%] (*n* = 49)	Non-mutant [%] (*n* = 536)	*p*-value
A > C or T > G	3.5	3.3	3.6	< 0.0001	3.9	3.4	0.2160
A > G or T > C	9.9	9.2	10.7	< 0.0001	9.6	9.9	0.7695
A > T or T > A	8.1	8.4	7.8	0.0005	8.6	8.1	0.4584
C > G or G > C	13.6	13.9	13.1	< 0.0001	13.2	13.6	0.3790
C > T or G > A	32.7	30.0	36.0	< 0.0001	28.6	33.0	0.5121
G > T or C > A	32.3	35.2	28.8	0.0001	36.0	32.0	0.4940

Technically, the workflows start with database queries for the TCGA barcodes of the patients with and without TP53 mutations. Subsequent queries obtain the overall survival of all patients, the overall size of genomic copy number aberrations in glioblastoma multiforme, and a list of all mutations in the cohort of patients with lung adenocarcinoma. These query results are stored as CSV files on the CancerSysDB server and are processed through workflow analysis scripts to restructure, analyze and visualize the data. The scripts for this TP53-dependent analysis of TCGA data were written in the functional statistics language R.

### Prediction of cancer types with random forests

In order to demonstrate the potential of our database for predictive analytics of clinically relevant traits, we have evaluated a workflow for the classification of a yet uncharacterized sample into one of the cancer types available in the CancerSysDB. This workflow can be applied, for instance, to predict the primary site of a tumor from a metastatic tissue specimen of unknown origin. The workflow is basically composed of two steps:

- In the *training phase,* a random forest consisting of 1000 trees is trained on all data available in the CancerSysDB. The workflow is composed of an HQL query with subsequent submission of the query results to a high-performance compute cluster. In order to control for the relatively strong imbalance in the class sizes, the workflow was implemented using a stratified sampling approach in the random forest training procedure. The random forest is then trained in 100 parallel processes with 10 trees in each process. Subsequently, the forest is loaded back into the CancerSysDB. The entire procedure must be repeated any time new data is being uploaded into the CancerSysDB. Random forests were chosen because of their good adaption to (binary) mutation data and their convenience in parallelization.
- In the *prediction phase*, a list of mutations of a yet unclassified sample can be uploaded into the CancerSysDB and is classified according to the random forest obtained in the training phase. As usual, the classification is determined by a majority vote between the 1000 classification trees in the forest.

**Fig. 2:**
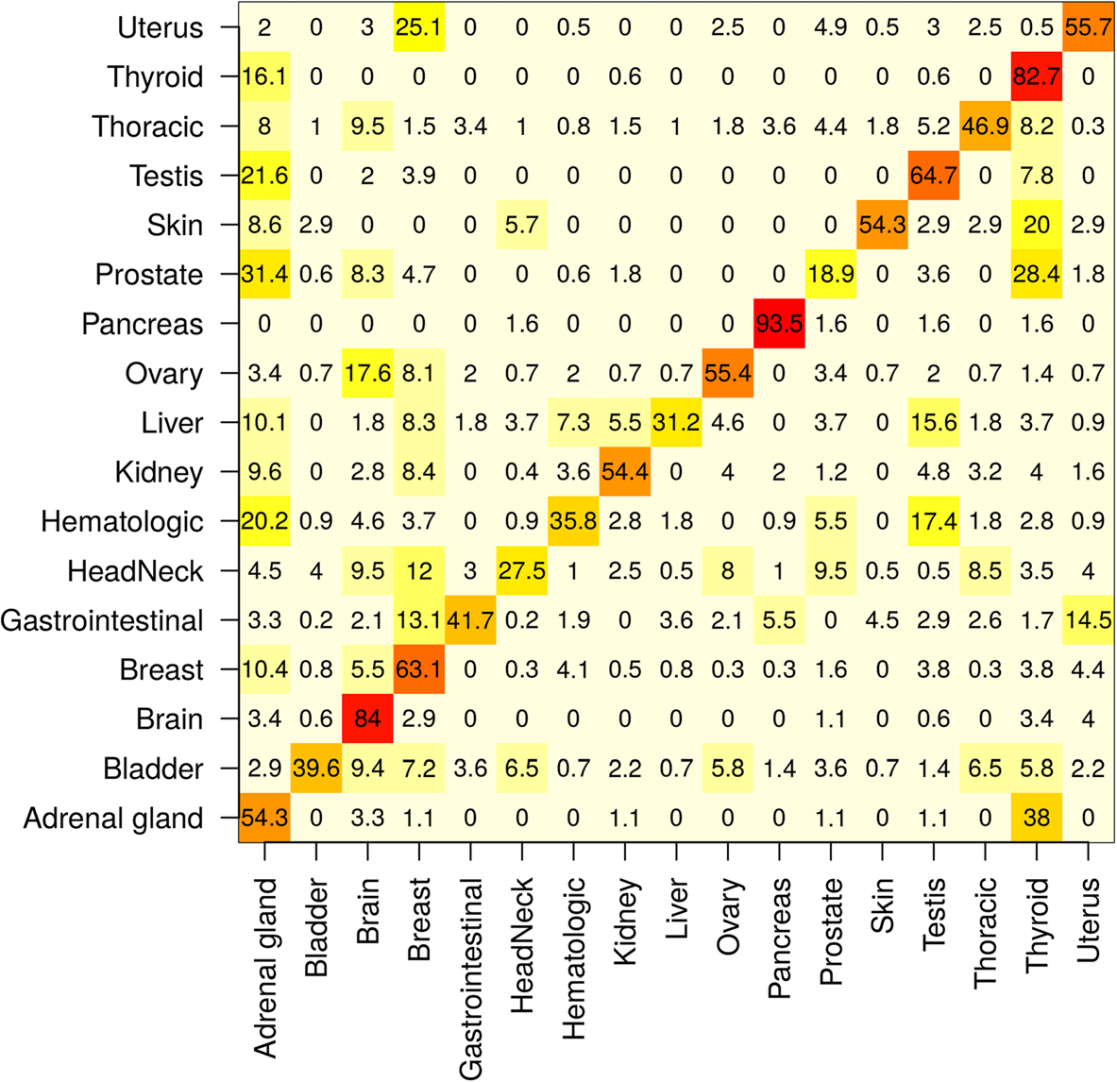
Results of a cross validation of the random forest prediction of cancer types in the CancerSysDB. The predictions are based on a random forest learned on the training set comprising 6006 patients from 30 TCGA studies (Table 2). Displayed are the predictions of the classes in the 3085 patients in the training set. The accuracy strongly varies across the particular subclasses, but sums up to a total of 1521 correctly classified patients (49.3%)

In the current workflow on the public instance, the training phase was carried out on data from 9091 patients in the CancerSysDB. To demonstrate that the predictions produced in this workflow are of sufficient accuracy to make them practically applicable, we split the 9091 patients in a training set of 6006 patients (66.6% in each cohort) and evaluated the predictions in a test set comprising 3085 patients (33.3% in each cohort; Table 3). Out of these 3085 patients in the test set, 1521 (49.3%) were assigned to the correct class (Fig. 2), whereas a random guess of the class would have produced a correct class assignment in only 182 cases (5.9%). Further evaluations of the workflow performance show that the success rate of the predictions does not increase with the number of trees nor the number of variables evaluated at each split, but strongly depends on the number of training samples (Additional file 2: Figure S1). In particular, Additional file 2: Figure S1c suggests that the accuracy could potentially be improved given a constantly growing amount of data in the CancerSysDB. However, we assume that the accuracy could be most stronly improved when including additional data types such as gene expression to the predictive algorithms.

**Table 3:** Classes of carcinomas used for random forest prediction of cancer types

Class name	TCGA cohorts	Sample size		
		Total	Training set	Test set
Adrenal gland	Adrenocortical carcinoma (ACC)	271	179	92
	Pheochromocytoma and paraganglioma (PCPG)			
Bladder	Urothelial carcinoma (BLCA)	411	272	139
Brain	Lower grade glioma (LGG)	515	340	175
Breast	Breast invasive carcinoma (BRCA)	1077	711	366
Gastrointestinal	Esophageal carcinoma (ESCA)	1237	817	420
	Stomach adenocarcinoma (STAD)			
	Colon adenocarcinoma (COAD)			
	Rectum adenocarcinoma (READ)			
	Cholangiocarcinoma (CHOL)			
Head & Neck	Head and neck squamous cell carcinoma (HNSC)	590	390	200
	Uveal melanoma (UVM)			
Hematologic	Acute myeloid leukemia (LAML)	321	212	109
	Diffuse large B-cell lymphoma (DLBC)			
	Thymoma (THYM)			
Kidney	Kidney Chromophobe (KICH)	738	488	250
	Renal clear cell carcinoma (KIRC)			
	Renal papillary cell carcinoma (KIRP)			
Liver	Hepatocellular carcinoma (LIHC)	321	212	109
Ovary	Ovarian serous cystadenocatcinoma (OV)	437	289	148
Pancreas	Pancreatic adenocarcinoma (PAAD)	184	122	62
Prostate	Prostate adenocarcinoma (PRAD)	498	329	169
Skin	Cutaneous melanoma (SKCM)	104	69	35
Testis	Testicular germ cell tumors (TGCT)	150	99	51
Thoracic	Lung adenocarcinoma (LUAD)	1143	755	388
	Lung squamous cell carcinoma (LUSC)			
	Mesothelioma (MESO)			
Thyroid	Thyroid carcinoma (THCA)	496	327	169
Uterus	Uterine carcinosarcoma (UCS)	598	395	203
	Uterine corpus endometrial carcinoma (UCEC)

### Analyzing TCA-cycle genes in kidney renal papillary cell carcinoma (KIRP)

We have implemented one interactive workflow, which allows users to perform an in-depth analysis of specific groups of genes or pathways. For the public instance of the CancerSysDB, we have chosen a set of mitochondrial functions. The interactive workflow consists of a bee swarm scatter plot displaying the differential expression (log2-fold change) of all genes in a selected pathway, as well as an interactive dashboard, where users can select the desired features for data display on the bee swarm scatter plot (see Additional file 3: Figure S2). Pathways to be shown can be selected on the right-hand side of the scatter plot. Features that can be chosen include the stage of the tumor, gender of the patients, as well as vital status. Differential expression is averaged over all individuals associated with a specific feature. If one feature is selected (e.g. stage of tumor) and the user hovers over any other fields of the dashboards, the data presented in the scatter plot are filtered accordingly. Hovering over one of the stages will give information on gender and vital status of all subjects within this stage (see for instance Additional file 3: Figure S2b, where hovering over Stage IV returns the information on gender (4 males) and vital status (3 alive, 1 dead) of all subjects of this tumor stage). Hovering over one of the other dashboards will change the data for averaging accordingly. For instance, when hovering over FEMALE, data are averaged over 10 patients in two stages (Stage I and Stage III), with 2 individuals with the vital status Dead and 8 ones with vital status Alive.

**Fig. 3:**
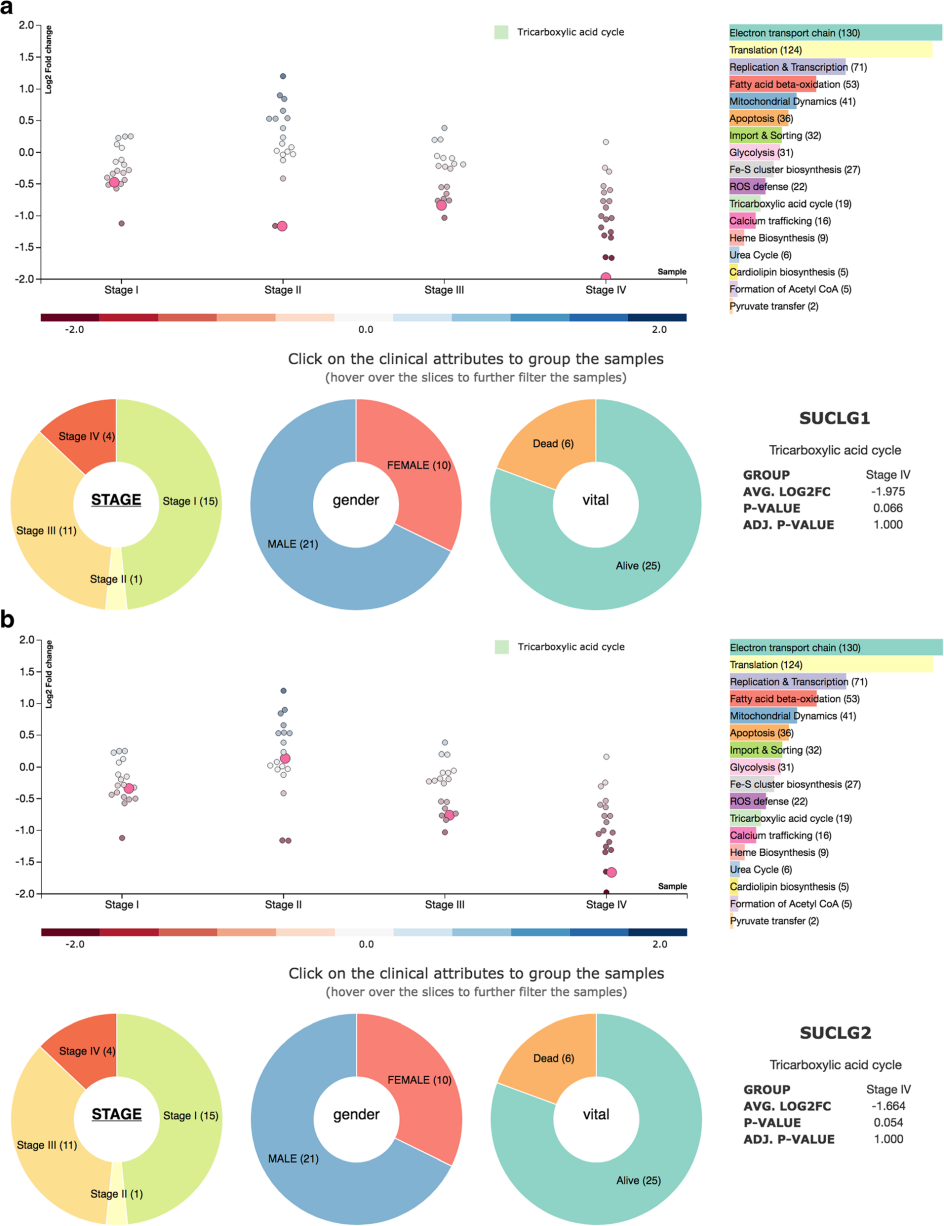
In-depth analysis of the dynamics of the TCA pathway in KIRP cancer patients. Interactive view bee-swarm scatter plot on the Tricarboxylic acid cycle (TCA) pathway from KIRP cancer patients is shown. The log2-fold changes are averaged for patients according to tumor grade (Stage I-IV). The dashboard gives the number of patients per grade and allows for further filtering according to gender or vital status (see also Additional file 2: Figure S1). **a** The SUCLG1 gene is selected (pink bubble in bee-swarm scatter plot). **b** The SUCLG2 gene is selected. Both genes show a strong, averaged down-regulation in Stage IV KIRP cancer patients (see Table 4 for averaged log2-fold changes)

We have used this workflow to observe the dynamics of the TCA pathway in KIRP (kidney renal papillary cell carcinoma) patients during tumor progression. We observed a strong down-regulation of the Succinate-CoA ligase subunits SUCLG1 and SUCLG2 in Stage IV KIRP patients (Fig. 3 and Table 4), which is independent of the vital status of the patients. We have not observed this specific down-regulation of both Succinate-CoA ligase subunits for any stage-specific cohort of any other tumor type imported from TCGA. An equally strong down-regulation of both subunits could only be observed for two sarcoma patients where no staging is done (SARC cohort in TCGA, data not shown).

**Table 4:** Averaged log2-fold changes of SUCLG1 and SUCLG2 mRNAs in different tumor stages of KIRP cancer patients

Stage	# Patients	Female/Male	Alive/Dead	SUCLG1		SUCLG2	
				log2 FC	*p*-value	log2 FC	*p*-value
I	15	5 / 10	13 / 2	−0.473	0.132	−0.338	0.307
II	1	0 / 1	1 / 0	−1.163	0.082	0.137	0.431
III	11	5 / 6	8 / 3	−0.835	0.018	−0.760	0.028
IV	4	0 / 4	3 / 1	−1.975	0.066	−1.664	0.054

Succinate-CoA ligase (SUCL) catalyses the conversion of succinyl-CoA and ADP or GDP to succinate and ATP or GTP. Substrate specificity is determined by the beta-subunit of the complex, which is either SUCLA2 (ATP) or SUGLG2 (GTP), while the alpha-subunit (SUCGL1) does not differ for either substrate [4]. SUCLG2 is predominately expressed in anabolic tissues such as liver or kidney [4, 5]; for these tissues, GTP is more important, as it is involved in processes such as gluconeogenesis or protein synthesis. Mutations of SUCLG1 lead to loss of SUCLG1 protein expression and subsequently to depletion of mtDNA; clinically, affected individuals suffer from severe acidosis and lactic aciduria [9]. Expression changes of SUCLG1 and 2 mRNA [2, 13], as well as protein [11, 17] were also identified in several studies as potential markers for kidney cancers. More notably, down-regulation of SUCLG2 protein levels are furthermore indicative for late stages in clear cell renal carcinomas [10].

## Conclusions

The CancerSysDB enables highly flexible analyses of cancer data across multiple OMICS data types and clinical data. We have demonstrated that the system can be used for cross-data type queries with clinically relevant information on prognosis, genome stability and mutation types of patients with and without mutations in the tumor suppressor TP53. In addition, we have given an example how machine learning technology on only one single data type (somatic mutations) can be used to achieve confident predictions of clinically relevant traits. Finally, we have provided an example how our system can be used as a platform for interactive analysis of different OMICS data types. The information provided by the TCGA data currently used in the public instance of the CancerSysDB is still very limited compare to the amount of data that can be expected in the near future when genomic analyses in a clinical context are becoming more and more a routine analysis. The CancerSysDB offers an appropriate framework to employ machine learning algorithms on much larger data volumes to predict, for instance, the overall survival of a patient and the response to a particular therapy given a patient’s molecular background.

## Additional files

Additional file 1: The source code of the database queries and workflow scripts for the three use cases reported in the paper. The results can be reproduced using the query results and analysis scripts provided. File query1.csv contains the barcodes of all samples for which mutation data do exist. File query2.csv contains the barcodes of all samples which carry a mutation in the gene of interest. Finally, query3.csv contains the survival data (according to Fig. 1a), a list of all mutations of patients in the cohort of interest (according to Fig. 1b), or a list of all genomic segments with aberrant copy number in the cohort of interest (according to Fig. 1c). There are small discrepancies between the number of patients with mutation data and the number of patients with survival data (Fig. 1a) and copy number data (Fig. 1c). (ZIP 4981 kb)

Additional file 2: **Figure S1** Overall success rate of the prediction of tumor types by random forests depending on (a) the number of samples per stratum in the random forest, (b) the number of variables picked randomly for each tree in the forest and (c) the number of trees learned in the forest. Importantly, the accuracy is increasing monotonically with the number of samples, indicating that the overall strategy is suitable, in particular, for a database with continuously growing amounts of data. In contrast, the success rate does not so much depend on the parameters chosen for the training phase of the random forest. (PNG 34 kb)

Additional file 3: **Figure S2** Interactive workflow of mitochondrial pathways. Shown is the Tricarboxylic acid cycle (TCA) pathway for KIRP cancer patients. The central view of this workflow is a bee-swarm scatterplot, which contains the averaged log2-fold changes of patient groups according to either tumor stage, gender or vital status. Each dot is represents the averaged log2-fold change of one gene that has been assigned to the chosen function. Functions can be selected on the right-hand side of the scatter plot. The dashboard below the scatter plot can be used to change the averaging according to a different feature ((a), which shows averaging according to stage), to display information on the composition of the selected feature ((b), which informs the user that all individuals of stage II, which was hovered over in this case, are male and that one individual is dead, while three of the patients are alive); or to further select individual patients and thus modify the averaging shown in the scatter plot ((c), where only female patients were chosen for stage-dependent averaging; as female patient data are only available for two stages (I and III), the scatter plot is changed accordingly). (PNG 679 kb)

## Abbreviations

API: Application Programming Interface
CBS: Circular Binary Segmentation
CSV: Character-separated variables
GDAC: Genome Data Analysis Center
GDC: Genomic Data Commons
HPC: High-performance computing
HQL: Hibernate Query Language
JSON: JavaScript Object Notation
KIRP: Kidney renal papillary cell carcinoma
MAF: Mutation annotation format
mRNA: Messenger ribonucleic acid
NGS: Next-Generation Sequencing
SARC: Sarcoma
TCGA: The Cancer Genome Atlas
