# Supplementary figures and images for: Integrative analysis and machine learning on cancer genomics data using the Cancer Systems Biology Database (CancerSysDB)
Source: BMC Bioinformatics. 2018 Apr 24;19:156. doi: 10.1186/s12859-018-2157-7 (PMC5921751; doi:10.1186/s12859-018-2157-7)

**Survival by mutation status (n=9444)**

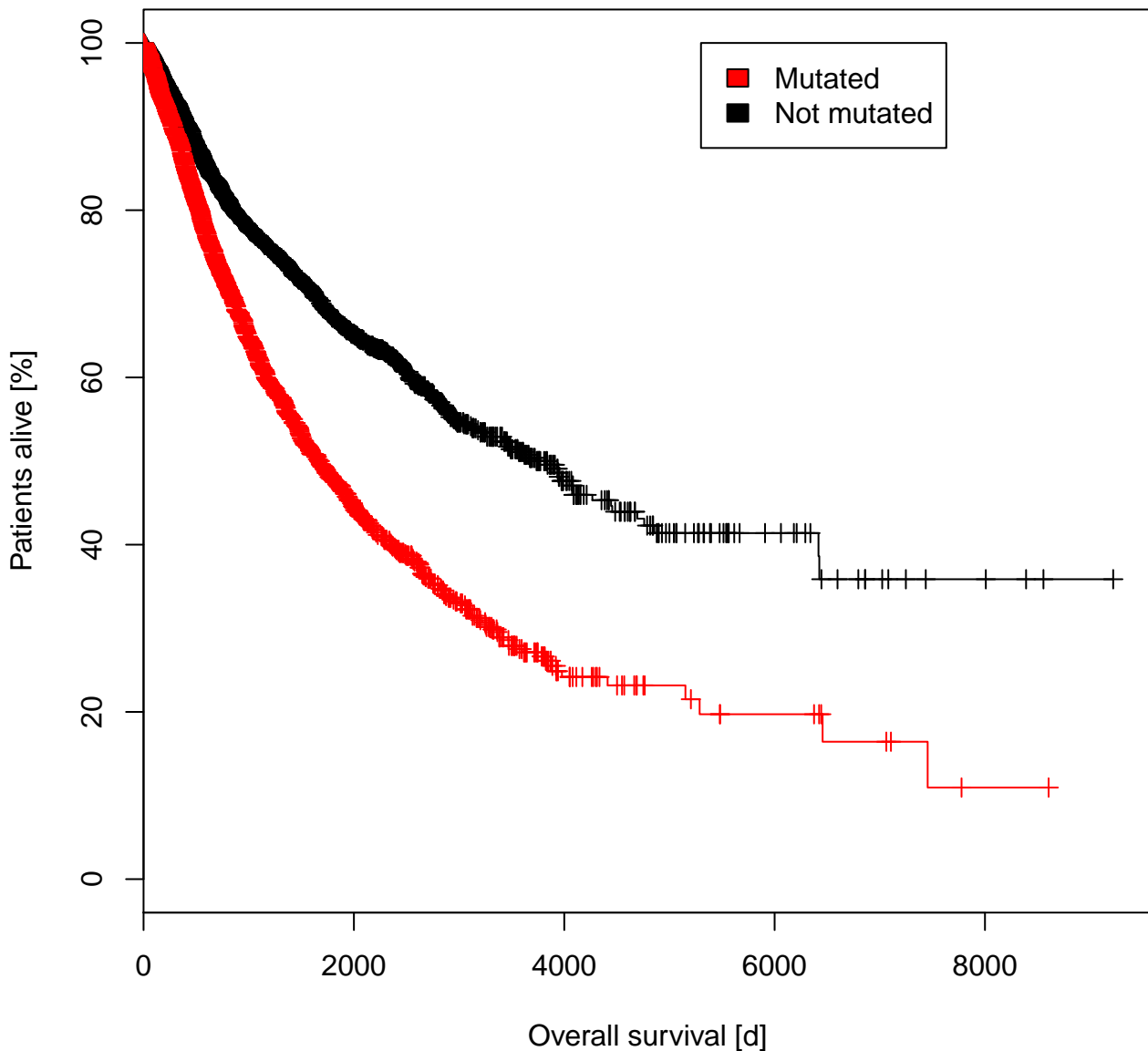

Supplement: Supplementary file 1 — The source code of the database queries and workflow scripts for the three use cases reported in the paper. The results can be reproduced using the query results and analysis scripts provided. File query1.csv contains the barcodes of all samples for which mutation data do exist. File query2.csv contains the barcodes of all samples which carry a mutation in the gene of interest. Finally, query3.csv contains the survival data (according to Fig. 1a), a list of all mutations of patients in the cohort of interest (according to Fig. 1b), or a list of all genomic segments with aberrant copy number in the cohort of interest (according to Fig. 1c). There are small discrepancies between the number of patients with mutation data and the number of patients with survival data (Fig. 1a) and copy number data (Fig. 1c). (ZIP 4981 kb) [file 12859_2018_2157_MOESM1_ESM.zip › QuerySource/Fig1a/showcase3_output.pdf]

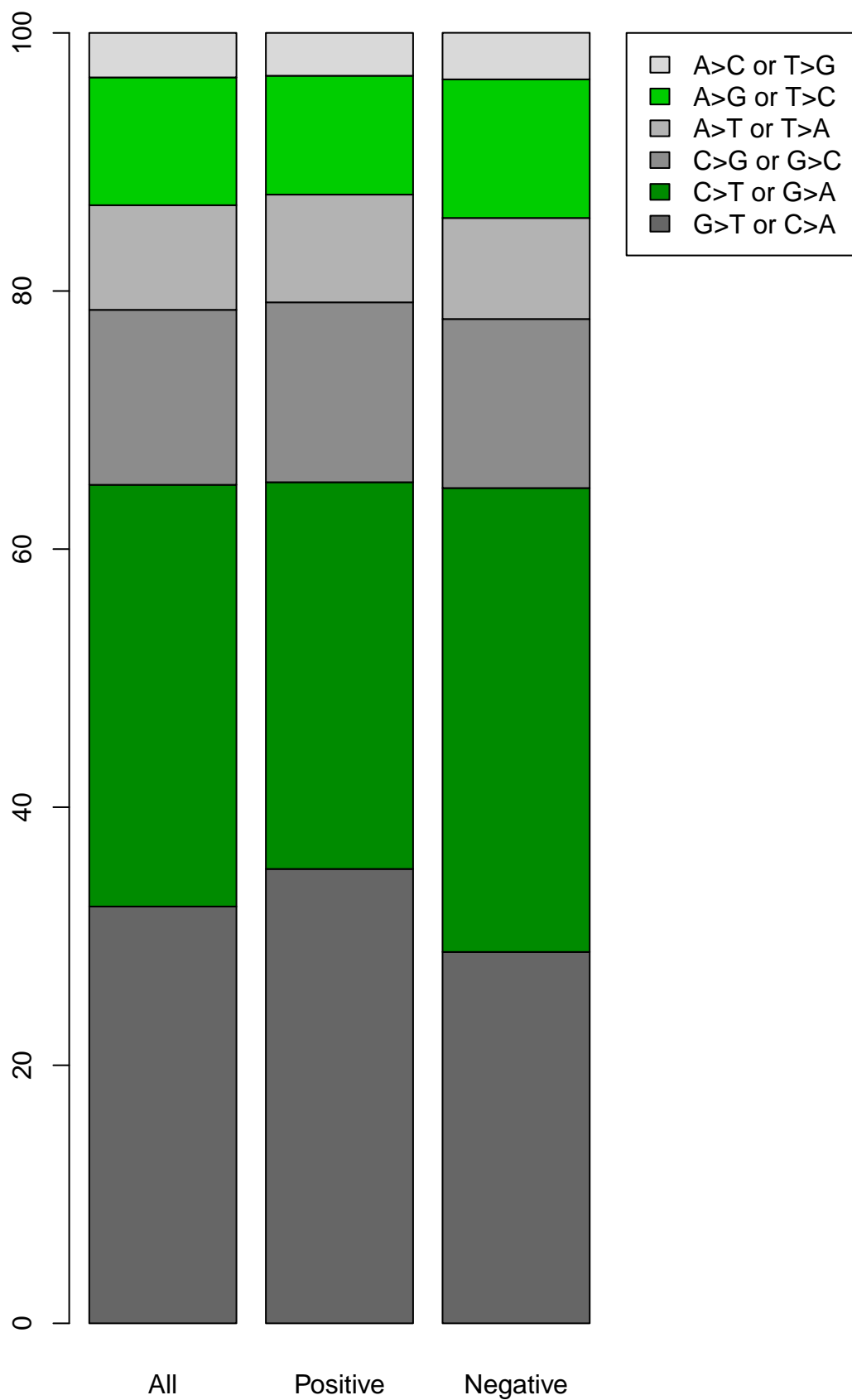

Supplement: Supplementary file 1 — The source code of the database queries and workflow scripts for the three use cases reported in the paper. The results can be reproduced using the query results and analysis scripts provided. File query1.csv contains the barcodes of all samples for which mutation data do exist. File query2.csv contains the barcodes of all samples which carry a mutation in the gene of interest. Finally, query3.csv contains the survival data (according to Fig. 1a), a list of all mutations of patients in the cohort of interest (according to Fig. 1b), or a list of all genomic segments with aberrant copy number in the cohort of interest (according to Fig. 1c). There are small discrepancies between the number of patients with mutation data and the number of patients with survival data (Fig. 1a) and copy number data (Fig. 1c). (ZIP 4981 kb) [file 12859_2018_2157_MOESM1_ESM.zip › QuerySource/Fig1b/VarTypes_output.pdf]

## Mutated

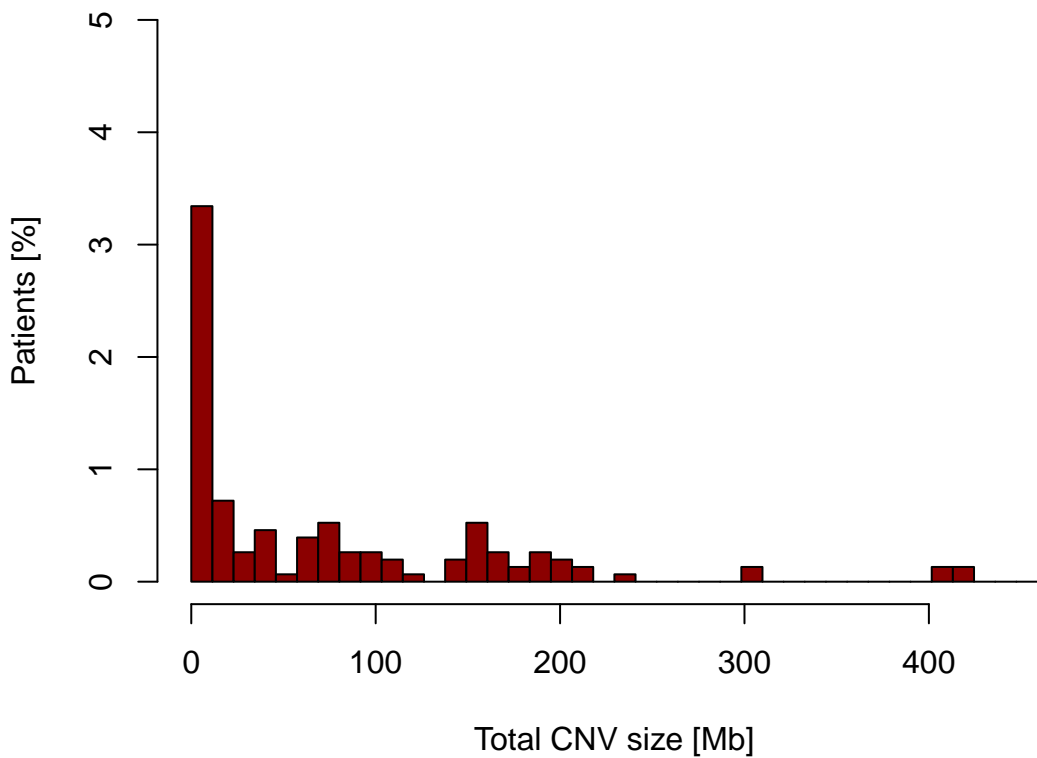

## Not mutated

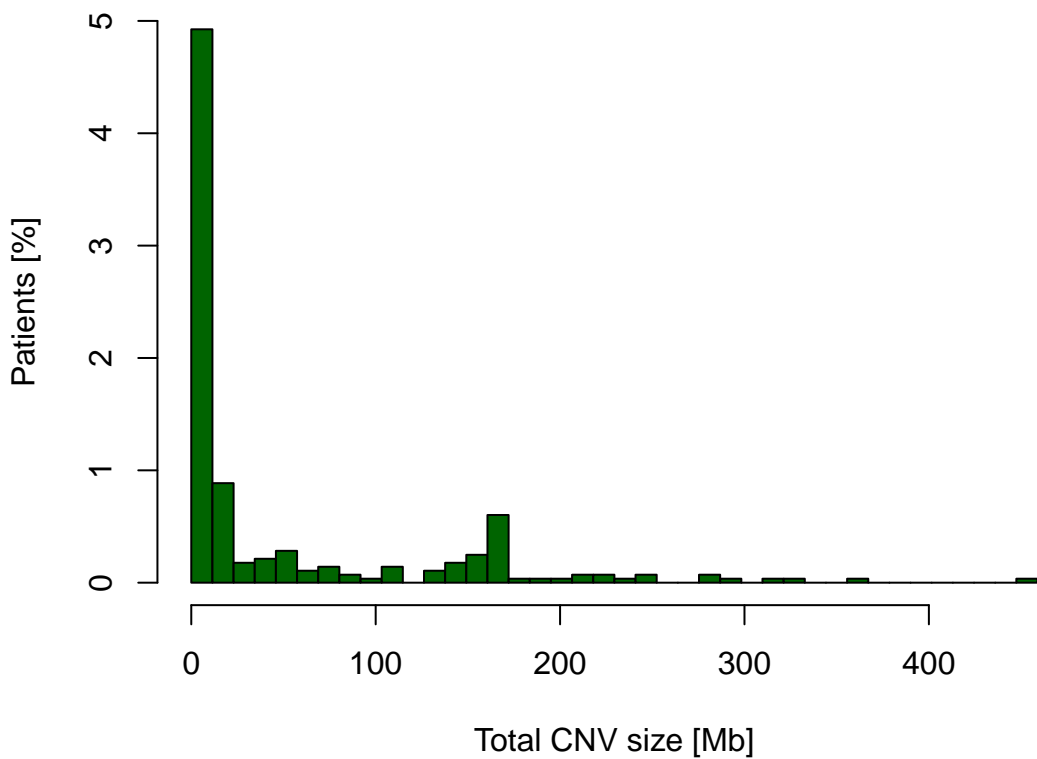

Supplement: Supplementary file 1 — The source code of the database queries and workflow scripts for the three use cases reported in the paper. The results can be reproduced using the query results and analysis scripts provided. File query1.csv contains the barcodes of all samples for which mutation data do exist. File query2.csv contains the barcodes of all samples which carry a mutation in the gene of interest. Finally, query3.csv contains the survival data (according to Fig. 1a), a list of all mutations of patients in the cohort of interest (according to Fig. 1b), or a list of all genomic segments with aberrant copy number in the cohort of interest (according to Fig. 1c). There are small discrepancies between the number of patients with mutation data and the number of patients with survival data (Fig. 1a) and copy number data (Fig. 1c). (ZIP 4981 kb) [file 12859_2018_2157_MOESM1_ESM.zip › QuerySource/Fig1c/CNAsize_output.pdf]

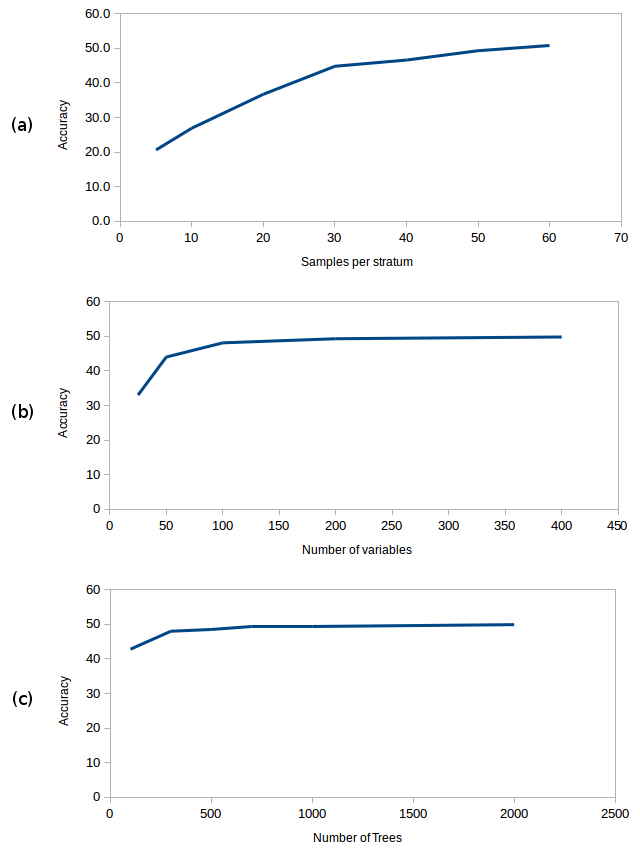

Supplement: Supplementary file 2 — Figure S1 Overall success rate of the prediction of tumor types by random forests depending on (a) the number of samples per stratum in the random forest, (b) the number of variables picked randomly for each tree in the forest and (c) the number of trees learned in the forest. Importantly, the accuracy is increasing monotonically with the number of samples, indicating that the overall strategy is suitable, in particular, for a database with continuously growing amounts of data. In contrast, the success rate does not so much depend on the parameters chosen for the training phase of the random forest. (PNG 34 kb) [file 12859_2018_2157_MOESM2_ESM.png]

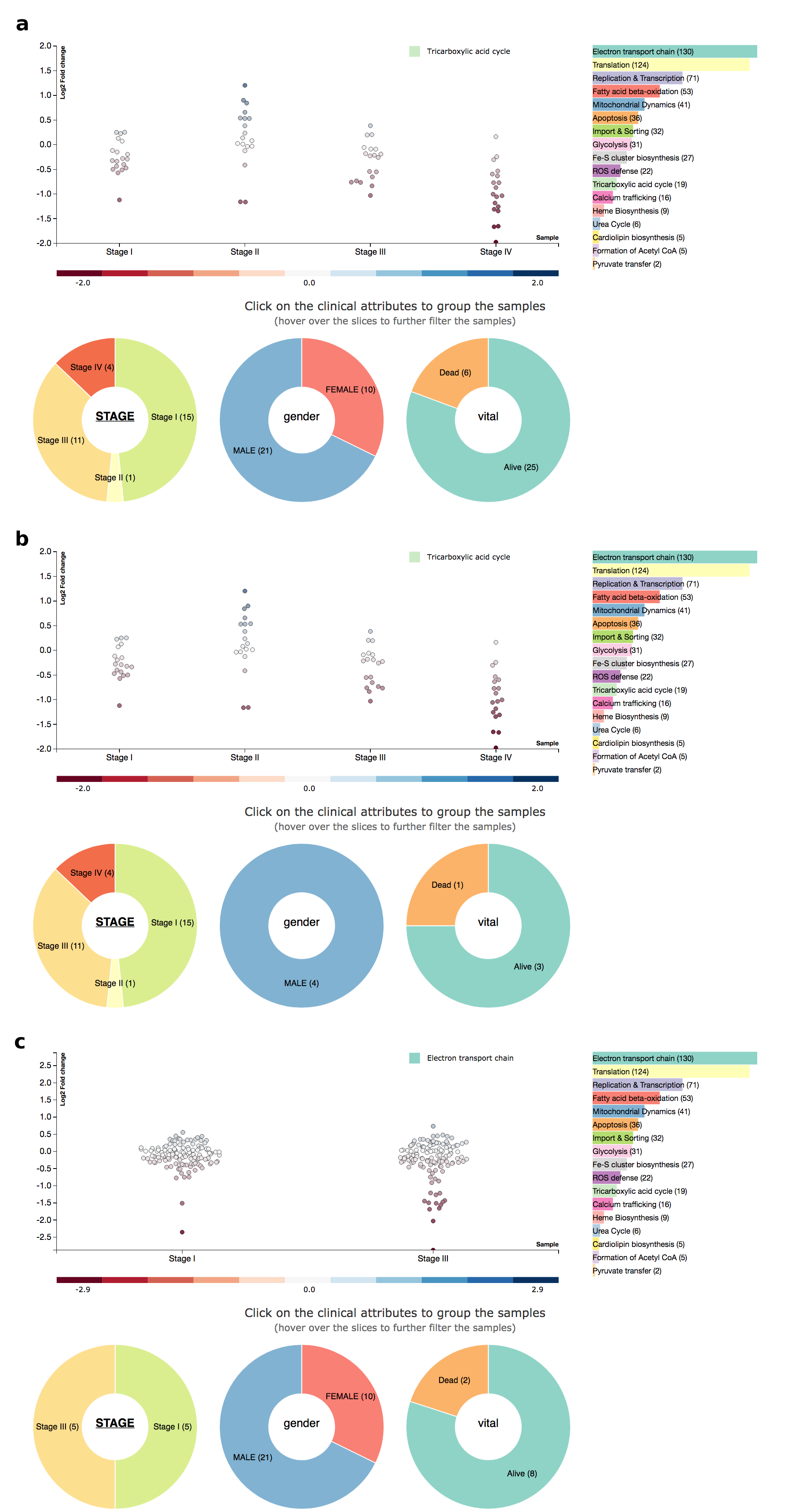

Supplement: Supplementary file 3 — Figure S2 Interactive workflow of mitochondrial pathways. Shown is the Tricarboxylic acid cycle (TCA) pathway for KIRP cancer patients. The central view of this workflow is a bee-swarm scatterplot, which contains the averaged log2-fold changes of patient groups according to either tumor stage, gender or vital status. Each dot is represents the averaged log2-fold change of one gene that has been assigned to the chosen function. Functions can be selected on the right-hand side of the scatter plot. The dashboard below the scatter plot can be used to change the averaging according to a different feature ((a), which shows averaging according to stage), to display information on the composition of the selected feature ((b), which informs the user that all individuals of stage II, which was hovered over in this case, are male and that one individual is dead, while three of the patients are alive); or to further select individual patients and thus modify the averaging shown in the scatter plot ((c), where only female patients were chosen for stage-dependent averaging; as female patient data are only available for two stages (I and III), the scatter plot is changed accordingly). (PNG 679 kb) [file 12859_2018_2157_MOESM3_ESM.png]
